# 

**Published:** 1918-08-03

**Authors:** 


August 3rd, 1918.] [Price Twopence

### The Hospital

The Workers' Newspaper of Administrative Medicine and Institutional Life, Administration, National Insurance and Health.

CONTENTS.

**Medical Reform and the Individual Patient** ... 379

**A Medical Witness on Medical Ethics and Things in General** ... ... ... ... ... 380

**Promotion in the Asylum Service** ... ... ... ... 385
The Greatest Hardship of the Assistant Medical Officer.

**Sophia Jex-Blake: A Great Personality** ... ... 387

She Opened the Medical Profession to Women.

**Parliament and the Profession** ... ... ... ... 386

**Hospital and Institutional News** ... ... ... 381
The Census of Medical Students—Haslar the Great—The British Science Guild—A Model Hospital Fair—A Medical Officer and Warrior Orphans—Proposed Federation of War Charities—Food Control and Asylum Death-rates—Lunatics as Attendants—The Longevity of Doctors—The Salary of Medical Superintendents—Safeguarding Posts for Absent Doctors—Complaints Against a Welsh Hospital Justified—Free Food for Poor Mothers—The Work of Sanatorium Patients—The Uninsured and Sanatorium Treatment—Higher Salaries at Worcester Infirmary—Lotteries for Charity—Two Methods of Appointment—Asylums Board Nurses at Buckingham Palace—The Dickens Home for the Blind—This Week's Drug Market.

CONTENTS.

**Life-Saving on the Thames** ... ... ... ... 388
More Rescue Stations Urgently Required.

**The Social Barometer** ... ... ... ... ... ... 391
From Samaritan Fund to Social Service Department.

**The Institutional Library** ... ... ... ... 389
A Medical Dictionary for the General Public.
The Author's Book his Everlasting Monument.

**Recent Official Publications** ... ... ... ... 398

**The Matrons', Sisters', and Women's Departments** 393
Some Experiences of Training-school Life.
Round the Hospitals.
The Fifth Year of the War—The Watchword of Nursing Service—The College's Three Studentships—£105 each—A Popular Nursing Leaflets Competition: Three Prizes Offered—Village Nurse Midwives.

**Her Majesty the Matron** ... ... ... ... 392

"We Call our Colonel 'Charlie,' and We Obey Orders."

**The London Hospital Nurses** ... ... ... ... ... 397

**The Editor's Letter-Box** ... ... ... ... 39

**A Hospital Meeting at Downing Street** ... ... 398

**Matrons' and Sisters' Appointments** ... ... ... 398

